# Thrombolysis with Systemic Recombinant Tissue Plasminogen Activator in Children: A Multicenter Retrospective Study

**DOI:** 10.4274/tjh.galenos.2021.2021.0038

**Published:** 2021-12-07

**Authors:** Emine Zengin, Nazan Sarper, Arzu Yazal Erdem, Işık Odaman Al, Melike Sezgin Evim, Neşe Yaralı, Burcu Belen, Arzu Akçay, Ayşen Türedi Yıldırım, Tuba Hilkay Karapınar, Adalet Meral Güneş, Sema Aylan Gelen, Hale Ören, Lale Olcay, Birol Baytan, Hüseyin Gülen, Gülyüz Öztürk, Mehmet Fatih Orhan, Yeşim Oymak, Sibel Akpınar, Özlem Tüfekçi, Meryem Albayrak, Burçak Tatlı Güneş, Aylin Canpolat, Namık Özbek

**Affiliations:** 1 Kocaeli University Faculty of Medicine, Department of Pediatrics, Division of Pediatric Hematology, Kocaeli, Turkey; 2 University of Health Sciences Turkey, Ankara City Hospital, Department of Pediatrics, Division of Pediatric Hematology, Ankara, Turkey; 3 University of Health Sciences Turkey, Dr. Behçet Uz Pediatrics and Pediatric Surgery Training and Research Hospital, İzmir, Turkey; 4 Uludağ University Faculty of Medicine, Department of Pediatrics, Division of Pediatric Hematology, Bursa, Turkey; 5 Başkent University Ankara Hospital, Clinic of Pediatrics, Division of Pediatric Hematology, Ankara, Turkey; 6 Acıbadem Mehmet Ali Aydınlar University Acıbadem Hospital, Clinic of Pediatric Hematology, İstanbul, Turkey; 7 Celal Bayar University Faculty of Medicine, Department of Pediatrics, Division of Pediatric Hematology, Manisa, Turkey; 8 Dokuz Eylül University Faculty of Medicine, Department of Pediatrics, Division of Pediatric Hematology, İzmir, Turkey; 9 Sakarya University Faculty of Medicine, Department of Pediatrics, Division of Pediatric Hematology, Sakarya, Turkey; 10 University of Health Sciences Turkey, Kanuni Sultan Süleyman Training and Research Hospital, Clinic of Pediatrics, Division of Pediatric Hematology, İstanbul, Turkey; 11 Kırıkkale University Faculty of Medicine, Department of Pediatrics, Division of Pediatric Hematology, Kırıkkale, Turkey; 12 University of Health Sciences Turkey, Tepecik Training and Research Hospital, Department of Pediatrics, Division of Pediatric Hematology, İzmir, Turkey; 13 İstanbul Medeniyet University Göztepe Training and Research Hospital, Clinic of Pediatrics, Division of Pediatric Hematology, İstanbul, Turkey

**Keywords:** Recombinant tissue plasminogen activator, Thrombolysis, Childhood thrombosis

## Abstract

**Objective:**

This study aimed to evaluate systemic thrombolysis experiences with recombinant tissue plasminogen activator (rtPA).

**Materials and Methods:**

Retrospective data were collected from 13 Turkish pediatric hematology centers. The dose and duration of rtPA treatment, concomitant anticoagulant treatment, complete clot resolution (CCR), partial clot resolution (PCR), and bleeding complications were evaluated. Low-dose (LD) rtPA treatment was defined as 0.01-0.06 mg/kg/h and high-dose (HD) rtPA as 0.1-0.5 mg/kg/h.

**Results:**

Between 2005 and 2019, 55 thrombotic episodes of 54 pediatric patients with a median age of 5 years (range: 1 day to 17.75 years) were evaluated. These patients had intracardiac thrombosis (n=16), deep vein thrombosis (DVT) (n=15), non-stroke arterial thrombosis (n=14), pulmonary thromboembolism (PE) (n=6), and stroke (n=4). The duration from thrombus detection to rtPA initiation was a median of 12 h (range: 2-504 h) and it was significantly longer in cases of DVT and PE compared to stroke, non-stroke arterial thrombosis, and intracardiac thrombosis (p=0.024). In 63.6% of the episodes, heparin was initiated before rtPA treatment. LD and HD rtPA were administered in 22 and 33 of the episodes, respectively. Concomitant anticoagulation was used in 90% and 36% of the episodes with LD and HD rtPA, respectively (p=0.0001). Median total duration of LD and HD rtPA infusions was 30 h (range: 2-120 h) and 18 h (2-120 h), respectively (p=0.044). Non-fatal major and minor bleeding rates were 12.5% and 16.7% for LD and 3.2% and 25.8% for HD rtPA, respectively. At the end of the rtPA infusions, CCR and PCR were achieved in 32.7% and 49.0% of the episodes, respectively. The most successful site for thrombolysis was intracardiac thrombosis. HD versus LD rtPA administration was not correlated with CCR/PCR or bleeding (p>0.05).

**Conclusion:**

Systemic thrombolytic therapy may save lives and organs effectively if it is used at the right indications and the right times in children with high-risk thrombosis by experienced hematologists with close monitoring of recanalization and bleeding.

## Abstract (tr)

**Amaç:**

Geriye dönük çok merkezli bir çalışma düzenleyerek, çocuk hematoloji uzmanlarının sistemik rekombinant doku plazminojen aktivatörü (rtPA) kullanımı ile ilgili deneyimlerinin ortaya konması amaçlandı.

**Gereç ve Yöntemler:**

Türkiye’deki 13 pediatrik hematoloji merkezinden geriye dönük veriler toplandı. rtPA tedavisinin dozu ve süresi, eşzamanlı antikoagülan tedavi, tam pıhtı erimesi (TPE), kısmi pıhtı erimesi (KPE) ve kanama komplikasyonları değerlendirildi. Düşük doz (DD) rtPA tedavisi 0,01-0,06 mg/kg/saat ve yüksek doz (YD) 0,1-0,5 mg/kg/saat olarak tanımlandı.

**Bulgular:**

2005-2019 yılları arasında ortanca yaşı 5 yıl (1 gün-17,75 yıl) olan 54 hastanın 55 trombotik epizodu değerlendirildi. Hastaların tanıları; intrakardiyak tromboz (n=16), derin ven trombozu (DVT) (n=15), inme dışı arteriyel tromboz (n=14), pulmoner tromboemboli (PE) (n=6) ve inme (n=4) idi. Trombüs saptanmasından rtPA başlangıcına kadar geçen süre medyan 12 saat (2 sa-504 sa) idi ve inme, inme olmayan arteriyel tromboz ve intrakardiyak tromboza kıyasla DVT ve PE için anlamlı olarak daha uzundu (p=0,024). Atakların %63,6’sında rtPA tedavisinden önce heparin başlandı. Atakların 22’sine DD ve 33’üne YD rtPA uygulandı. DD ve YD rtPA ataklarının sırasıyla %90’ında ve %36’sında eş zamanlı antikoagülasyon kullanıldı (p=0,0001). DD ve YD rtPA infüzyonlarının medyan toplam süresi sırasıyla 30 sa (2 sa-120 sa) ve 18 sa (2 sa-120 sa) idi (p=0,044). Ölümcül olmayan majör ve minör kanama oranları DD için sırasıyla %12,5, %16,7 ve YD rtPA için %3,2, %25,8 idi. rtPA infüzyonlarının sonunda, atakların sırasıyla %32,7’sinde TPE ve %49’unda KPE elde edildi. Tromboliz için en başarılı bölge intrakardiyak trombozdu. YD’ye karşı DD rtPA uygulaması, TPE/KPE veya kanama ile korele değildi (p>0,05).

**Sonuç:**

Yüksek riskli trombozlu çocuklarda sistemik trombolitik tedavi, deneyimli hematologlar tarafından rekanalizasyon ve kanamanın yakın takibi altında doğru endikasyon ve doğru zamanda kullanıldığında etkin bir şekilde hayat ve organ kurtarabilir.

## Introduction

The incidence of pediatric thrombosis is increasing due to advancements in neonatal and pediatric intensive care. Central venous catheters (CVCs), infections, surgery, immobility, trauma, congenital heart disease (CHD), vasculitis, underlying cancer, and thrombophilia are important risk factors for thrombosis [1,2,3].Endovascular and surgical interventions for CHD save many lives, but the development of thrombosis is a frequent complication of these procedures [4]. Anticoagulation with unfractionated heparin (UFH) or low-molecular-weight heparin (LMWH) is the routine management of pediatric acute thrombosis. Heparins are used to prevent propagation, embolization, and recurrence of acute thrombosis [5].Anticoagulation does not provide rapid recanalization of the occluded vessels and 49% of surviving infants and children suffering from arterial thrombosis have long-term sequelae including extremity or organ loss or epilepsy [1,3]. Postthrombotic syndrome (PTS) is the most common long-term complication of deep vein thrombosis (DVT) of the extremities. It is a chronic condition characterized by the development of venous insufficiency that manifests clinically with swelling, pain, cramping, stasis, dermatitis, and ulceration of the affected extremity [6]. The incidence of pediatric PTS ranges from 3% to 70% in median 2-year follow-up [6,7]. In cases of pediatric arterial thrombosis and DVT, mortality is nearly 4-9% and 2%, respectively, with cranial and intracardiac thrombosis causing the most mortality [1,8]. Since 1990, off-label use of recombinant tissue plasminogen activator (rtPA) in newborns and children has shown that systemic thrombolytic therapy is very effective if it is used at the right indications in selected patients under close monitoring [9,10,11,12,13,14]. Endovascular thrombolysis requires expert interventional radiologists and general anesthesia, and the small vessel diameters of children frequently limit such procedures; in addition, this intervention may damage the vascular endothelium and induce new thrombosis. However, the intravenous administration of thrombolytics is practical [5]. In this retrospective study, the experiences of pediatric hematology centers with systemic thrombolysis are presented. This study may help in the timely referral of pediatric patients to hematologists, intensive care physicians, or cardiologists for thrombolysis in cases of organ-, limb-, and life-threatening thrombi.

## Materials and Methods

This retrospective study was approved by the institutional ethics committee and approval was obtained from the patients’ legal guardians at admission to the hospital for the release of medical data for scientific purposes without including patient identity. A questionnaire form was designed and submitted to all Turkish pediatric hematology centers via electronic mail to collect the medical data of patients 0-18 years old who received systemic rtPA for thrombolysis. rtPA administrations for CVC clearance were not included in the study. Major surgery or central nervous system bleeding within 10 days and inability to maintain a platelet count of at least 50,000/µL with transfusion support or fibrinogen of at least 100 mg/dL were exclusion criteria. Concomitant heparin administration was at the decision of the attending physician. UFH dose of 5-10 U/kg/h and LMWH (enoxaparin) dose of 0.5 mg/kg twice daily were the prophylactic doses. Patients’ age, sex, underlying disease, location of the thrombus, period from thrombus detection to rtPA administration, previous anticoagulant treatments, initial and maximum rtPA doses, concomitant anticoagulant treatments, total duration of rtPA infusions, bleeding complications, thrombus resolution, amputations, organ loss, and outcomes after 3 months of anticoagulation were evaluated. Low-dose (LD) rtPA treatment was defined as 0.01-0.06 mg/kg/h and high-dose (HD) as 0.1-0.5 mg/kg/h [5,15]. The centers reported that for older children and adolescents receiving LD continuous infusion, the maximum dose per hour was not defined and rtPA doses exceeding 2 mg/h were administered. Major bleeding was defined as any intracranial or retroperitoneal bleeding, or bleeding resulting in a drop in hemoglobin of 2 g/dL, or requiring transfusion or leading to death as defined by the International Society on Thrombosis and Haemostasis bleeding scale [16]. Minor bleeding was defined as oozing at the site of intravenous or other indwelling catheters or minor epistaxis, hematoma, hematuria, or melena not resulting in a drop in hemoglobin.

Diagnosis of thrombosis and monitoring for clot resolution performed by color Doppler ultrasound examination, echocardiography, and/or computed tomographic angiography was acceptable. Complete clot resolution (CCR) was defined as objective radiologic imaging confirming recanalization of the occluded vessel and/or arterial thrombosis clinical examination showing equal pulse pressure, capillary return, and warm extremities. Partial clot resolution (PCR) was defined as objective radiologic imaging confirming partial recanalization of the occluded vessels. No resolution (NR) was defined as objective radiologic imaging confirming no change or thrombus extension in the occluded vessel.

Centers reported performing laboratory monitoring by measuring prothrombin time, activated partial thromboplastin time, fibrinogen, anti-factor Xa, fibrin degradation products, D-dimer, platelet counts, and hemoglobin levels every 6-12 h. Patients were strictly monitored clinically for bleeding (oozing from puncture sites, gastrointestinal system bleeding, and signs of cerebral bleeding), any new emboli, allergic and anaphylactoid reactions, laryngeal edema, orolingual angioedema, rash, and urticaria. Centers also monitored blood pressure, heart rate, and respiration rate. If minor bleeding developed, the rtPA dose was either deescalated or rtPA was withdrawn transiently. They stopped the treatment and infused fresh frozen plasma (FFP) and/or red blood cells if major bleeding developed. If recanalization was seen in imaging studies, rtPA was stopped.

### Statistical Analysis

For calculation of median and percentage, descriptive analysis was used; for comparison of two independent variables, the Mann-Whitney U test was used; and for comparison of more than two independent variables, Kruskal-Wallis H tests were used. Values of p<0.05 were defined as significant. All analyses were performed using SPSS version 13 (SPSS Inc., Chicago, IL, USA).

## Results

Thirteen pediatric hematology centers participated in the study. Questionnaire forms for patients aged 0-18 years who received systemic rtPA (alteplase) between May 2005 and December 2019 were evaluated. The study included 55 thrombotic episodes of 54 patients (24 male, 30 female) with a median age of 5 years (range: 1 day to 17.75 years). Two episodes (intracardiac and diffuse pulmonary thromboembolism) of a patient with CHAPLE syndrome were included. Eight of these cases were already published [11,17]. Table 1 shows the characteristics of patients and treatment outcomes. Patients with intracardiac and non-stroke arterial thrombosis were younger than patients with pulmonary thromboembolism, DVT, and stroke (p=0.001). 

Out of 16 patients with intracardiac thrombus, five were preterm babies in the neonatal intensive care unit. They had umbilical catheters and ventilator support, and two of them also had septicemia. Five patients had CHD (one of them also had leukemia) and another two patients had cardiomyopathy. There were also patients with chronic pulmonary disease in the intensive care unit, acute lymphoblastic leukemia, lipin-1 deficiency (rhabdomyolysis + acute renal failure with hemodialysis), and CHAPLE syndrome (CD55 deficiency).

Out of 15 patients with DVT, five had hematological diseases (all with CVCs) and three had metabolic diseases (propionic academia with CVC and *Candida* septicemia, hyperhomocysteinemia, and hyperlipidemia). There were also cases of Behçet’s disease, pyelonephritis, exposure to trauma during football activity, Down syndrome, epileptic seizures, idiopathic Budd-Chiari syndrome, and a patient taking oral contraceptive pills.

In 14 patients with non-stroke arterial thrombosis, the underlying diseases were prematurity-associated in five patients (septicemia, respiratory distress syndrome, necrotizing enterocolitis, bronchopulmonary dysplasia) and one baby also had CHD. They also had umbilical catheters as a thrombus-provoking factor. CHD was also present in another four patients: Fallot tetralogy, ventricular septal defect (VSD) + patent foramen ovale + pulmonary hypertension, a newborn with aortic stenosis who underwent cardiac catheterization, and an infant with VSD who underwent transcatheter repair. Other patients had Kawasaki syndrome with coronary artery thrombus and pulmonary stenosis (n=2), while there was also an infant with bronchiolitis in the pediatric intensive care unit and an adolescent in the postpartum period with inherited homozygous antithrombin (AT) deficiency.

In cases of pulmonary thromboembolism, underlying diseases were nephrotic syndrome, immune dysregulation (Evans syndrome; the patient received corticosteroid), Behçet’s disease, multiple sclerosis with corticosteroid treatment, CHAPLE syndrome (CD55 deficiency), and obesity and pregnancy in a 17-year-old patient.

Underlying diseases of the patients with stroke were hematological disease in two (leukemia; a female adolescent with aplastic anemia who underwent hematopoietic stem cell transplantation, having a CVC and receiving depot progesterone), infection in one (acute mastoiditis), and idiopathic thrombus in a toddler.

As provoking factors for thrombus, there were CVCs, infections, and malignancy in 20 (36.4%), 10 (18.2%), and 7 (12.7%) of the thrombotic episodes, respectively. There was more than one thrombotic risk factor for some patients (Table 1).

In 63.6% (35/55) of the episodes, heparin was initiated before rtPA treatment. LMWH was preferred in 33 episodes and UFH in only two episodes. Six patients received FFP to replace plasminogen before administration of rtPA; two of them were infants. The period from thrombus detection to rtPA initiation was a median of 12 h (2-504 h); this period was significantly longer for DVT and pulmonary thromboembolism compared to stroke, non-stroke arterial, and intracardiac thrombi (p=0.024). When all 55 episodes were evaluated, no correlation was found between this period and thrombus resolution (p=0.75).

There was a wide range of administered rtPA doses and durations. In 14 episodes, dose escalation, in four episodes dose de-escalation, and in two episodes temporary withdrawal of rtPA due to bleeding was applied. The median initial and median maximum rtPA doses were both 0.1 mg/kg/h (0.01-0.5). The total duration of rtPA infusions was a median of 18 h (2-120 h) and it was significantly shorter for cardiac thrombosis compared to pulmonary and arterial thrombosis (p=0.015).

In this study, 33 patients received HD rtPA. Only one center used a standard rtPA treatment protocol. They used HD rtPA for 6 h and repeated infusion after 24 h if thrombus resolution was not achieved. They also transfused FFP at 10-20 mL/kg concomitantly. They did not use concomitant heparin. This center had five patients in this study. For four patients, they had to repeat rtPA infusions 3-6 times (totally for 18-36 h) and reported no bleeding complications. One of their patients with femoral and popliteal artery thrombus had CCR after only 6 h of rtPA infusion. Out of their five patients (two cardiac, one peripheral artery, one pulmonary thromboembolism, one DVT), two achieved CCR. Eight more patients from other centers received HD rtPA for only 2-6 h. When all patients receiving HD rtPA for ≤6 h (total duration) were evaluated, 2/9 achieved CCR with two minor bleeding complications. Patients receiving HD rtPA for 6 h at a time with or without repeated daily doses had two minor bleeding complications (2/13) and only two had CCR (2/13).

In Table 2, patients receiving HD rtPA infusion for longer than 6 h/day are presented. Patients 5, 6, and 16 received rtPA for 7-12 h daily whereas the others received rtPA without interruption. These 20 patients received HD rtPA for 12-120 h totally and ten achieved CCR; they had one major and six minor bleeding complications. In eight episodes, concomitant heparin was used. FFP was used in only two episodes. The numbers were small for statistical comparison of the infusion period concerning thrombus resolution and bleeding complications. In HD rtPA treatment, a longer infusion period compared to ≤6 h seemed more effective (CCR 10/20 versus 2/9), but with more bleeding complications (six minor and one major bleeding episode in 20 patients versus two minor bleeding episodes in 9 patients).

Concomitant heparin was administered in 58.2% (32/55) of the thrombotic episodes (10 UFH and 22 LMWH), in 24 episodes at the therapeutic dose and in eight episodes at the prophylactic dose. Therapeutic-dose heparin was preferred in patients receiving LD rtPA (p<0.05). In intracardiac thrombosis, concomitant anticoagulant administration was less frequent compared to stroke or DVT (p=0.046). In 18 episodes of this study, CCR was achieved, and in 11 of those, concomitant heparin was not used. In 10 of these episodes, the thrombi were intracardiac.

Four major non-fatal bleedings (7.2%) developed; three were in patients with pulmonary thromboembolism (pulmonary hemorrhage, melena + hematemesis, hematuria) and one in a patient with coronary artery thrombosis (hematuria). Underlying diseases in the patients developing major bleeding were Kawasaki syndrome, Behçet’s disease, Evans syndrome, and multiple sclerosis; they all received concomitant heparin and three of them received LD rtPA for 18-48 h. FFP and packed red cells or only FFP or fibrinogen concentrate were administered as supportive treatment in major bleeding episodes. Antifibrinolytics were not used for any patient. There were minor bleedings in 12 episodes (21.8%) that could be easily controlled; these were epistaxis, gingival bleeding, venipuncture site bleeding, CVC exit site or incision site bleeding, subcutaneous hematoma, and bone marrow puncture site bleeding. When patients with and without rtPA-related bleedings were compared, no relationship was found with age, gender, initial rtPA dose, maximum rtPA dose, site of thrombosis, total rtPA duration, or concomitant use of anticoagulants. In 22 patients receiving LD treatment, there were three major and four minor bleeding complications. In patients receiving LD continuous treatment, rtPA doses exceeding 2 mg/h were used in older children and adolescents. When major bleedings in patients receiving LD were evaluated, a dose above 2 mg/h was administered for only one adolescent with pulmonary thromboembolism. This patient received concomitant LMWH and had hematuria and injection site bleeding. Two other patients on LD treatment and with major bleeding also received concomitant LMWH.

As shown in Table 1, after rtPA administration, CCR and PCR were achieved in 18 (32.7%) and 27 (49.0%) of the episodes, respectively. In intracardiac thrombosis, CCR was achieved in 62.5% and PCR in 37.5%; thrombolysis was significantly more successful compared to patients with non-stroke arterial thrombosis (CCR 21% and PCR 42%) and patients with DVT (CCR 13.3% and PCR 60%) (p=0.006). Out of 16 patients with intracardiac thrombosis, CCR was achieved in two patients even with late rtPA administrations (7 and 21 days after diagnosis). The total duration of rtPA infusion was significantly shorter in intracardiac thrombosis compared to pulmonary thromboembolism and non-stroke arterial thrombosis. Physicians discontinued treatment when CCR was achieved. Two patients achieving only PCR after systemic thrombolysis with rtPA underwent endovascular thrombectomy: a patient with Kawasaki syndrome having coronary artery thrombus and another patient with left iliac and femoral vein thrombosis. The latter patient also developed a thrombus in the contralateral extremity in the following week under LMWH treatment. rtPA was administered again but due to NR with the second thrombus, endovascular thrombectomy was performed. Off-label rivaroxaban was started with special approval from the health authority.

There were two patients with unilateral renal artery thrombosis. The patient with inherited AT deficiency presented with left renal artery thrombosis in the postpartum period and left kidney atrophy developed. A 3.5-year-old patient with preexistent right kidney atrophy presented with left renal artery thrombosis and achieved CCR. There was an 11-year-old patient with pyelonephritis associated with left renal vein thrombosis (RVT); rtPA treatment was started 7 days after the diagnosis and PCR was achieved. This patient also already had right kidney atrophy at presentation. Unilateral blindness developed in a patient after stroke; the etiology of the thrombus was otitis media and mastoiditis. Amputations were performed for three patients with arterial thrombosis of extremities or fingers after unsuccessful thrombolytic treatments. Cirrhosis developed in a patient with idiopathic Budd-Chiari syndrome; this patient underwent liver transplantation. Three patients died due to prematurity complications of septicemia + necrotizing enterocolitis + bronchopulmonary dysplasia and one transplanted patient with juvenile myelomonocytic leukemia died due to veno-occlusive disease. Anticoagulant treatments were continued with LMWH for a median of 3 months (1-6 months) in all surviving patients except three patients who received coumadin and rivaroxaban and one non-compliant patient. Acetylsalicylic acid was also used for seven patients with arterial, cardiac, and venous thrombosis. The patient with CHAPLE syndrome also received eculizumab. When the patients’ outcomes were evaluated after 3 months of anticoagulation, CCR was increased to 74.5% from 32.7%.

In Table 3, LD and HD rtPA treatments are compared in terms of characteristics of thrombotic episodes, bleeding complications, and treatment outcomes. Patients’ median age and median duration from thrombus detection to rtPA administration were similar between patients receiving LD and HD treatment. Although the numbers were small for statistical power, there were more patients with intracardiac thrombosis in the HD group. In the LD group, the median duration of rtPA treatment was longer (p=0.044) and concomitant anticoagulant administration was more frequent (p=0.0001). In LD treatment 90% (20/22) and in HD treatment 36% (12/33) of the patients received concomitant anticoagulants. The dose of rtPA was not correlated with clot resolution or bleeding (p>0.05).

## Discussion

The lack of prospective trials for the treatment of pediatric thrombosis with rtPA is a serious dilemma for pediatricians. The Thrombolysis in Pediatric Stroke Study started in 2010 but was ended by the National Institutes of Health in 2013 due to lack of accrual [18]. In the pediatric antithrombotic therapy guidelines published in 2012, thrombolysis was recommended for the following indications [19]: a) limb-threatening or organ-threatening arterial thrombosis (via proximal extension) or femoral artery thrombosis that fails to respond to initial UFH therapy; b) bilateral RVT with evidence of renal impairment; c) symptomatic peripheral arterial catheter-related thromboembolism; d) right atrial thrombosis related to CVC, especially >2 cm and mobile; and e) thrombotic giant coronary artery aneurism in cases of Kawasaki syndrome. The authors were more cautious in thrombolysis of childhood acute ischemic stroke outside of research protocols [19]. Some authors also included the following conditions among strong indications for thrombolysis: superior vena cava syndrome; pulmonary embolism with hypotension or shock or resulting in right heart strain or myocardial necrosis; extensive venous thrombosis with total occlusion of venous flow, increased compartment pressures, and compromise of arterial blood flow; and CHD with shunt thrombosis and cerebral sinovenous thrombosis with neurological impairment and no improvement with anticoagulation or progressive thrombosis [5].

Tarango and Manco-Johnson [5] recommended systemic thrombolysis with LD treatment for 6-72 h and HD treatment for 2-6 h at a time, repeating the same doses if indicated over a period of 72 h. In the current retrospective study, only one center applied HD treatment following this protocol, administering rtPA for 6 h at a time and repeating the infusion every 24 h if there was no clot resolution. However, they repeated the infusions for 6 days for some patients, longer than recommended by Tarango and Manco-Johnson [5]. Other centers used continuous infusions of LD and HD rtPA for a maximum of 120 h or stopped infusions earlier when CCR was achieved or bleeding complications occurred. When HD rtPA infusions for longer than 6 h/day were compared to HD for 2-6 h/day, bleeding complications were more frequent in infusions for >6 h (six minor bleedings and one major bleeding in 20 thrombotic episodes versus two minor bleedings in 13 thrombotic episodes). However, with LD continuous infusion (2-120 h), there were also four minor and three major bleeding episodes in 22 thrombotic episodes. In this series, major bleeding was more frequent with LD rtPA compared to HD rtPA. Centers administered continuous LD without any dose limitation per hour, exceeding 2 mg/h in older children and adolescents. Although bleeding episodes were non-fatal in this retrospective study, to be on the safe side, administration of continuous LD rtPA for longer than 72 h and/or exceeding 2 mg/h cannot be recommended, similar to administration of HD rtPA for longer than 6 h at a time.

Similar to our study, LD rtPA was shown to be effective and safe in a group of patients including preterm neonates and children [15]. In the present study, when LD and HD rtPA treatments were compared, there was no difference in clot resolution, but with LD rtPA, the median duration of treatment was longer and physicians preferred concomitant heparin administration more frequently. This may be explained by physicians’ fear of bleeding complications in HD treatment. CCR was achieved in 32.7% and PCR in 49.0% of the thrombotic episodes at the end of rtPA infusions. Similar to our results, in a study of 46 children receiving systemic rtPA, Ansah et al. [12] found that CCR and PCR/NR were not related to initial and maximum rtPA doses, duration of rtPA, or mean time from diagnosis to treatment. They reported that CCR and PCR were achieved in 46% and 22%, respectively, with a slightly higher CCR rate than that seen in our study. They also reported that bleeding complications occurred in 33% of the patients and bleeding-related death in 4.3%, and bleeding complications were related to median initial rtPA dose (0.10 mg/kg/h vs. 0.03 mg/kg/h) [12]. In the present study, in 63.6% of the episodes, heparin was initiated before rtPA treatment and concomitant heparin was administered to 90% of the patients receiving LD rtPA. Concomitant heparin was used to prevent proximal clot extension during rtPA infusion. The median duration of rtPA treatment was 30 h (range: 2-120 h) with LD treatment and 18 h (2-504 h) with HD. In Wang et al.’s [15] study, the duration of treatment was 4-48 h with LD and 1-24 h with HD; complete lysis was achieved in 97% of 29 patients with arterial thrombus, intracardiac thrombus, and DVT presented within 2 weeks. It is not easy to compare these studies and draw any conclusions due to the heterogeneity of the patients.

In the present series, early CCR and PCR rates were between 0% and 62.5% and between 37.5% and 75.0%, respectively, for different thrombus locations, but thrombolysis was significantly more successful for intracardiac thrombi and pulmonary thromboembolism (62.5% and 50% CCR, respectively). Fortunately, after two endovascular thrombectomies following unsuccessful systemic rtPA administrations, and with 3 months of anticoagulation, CCR ranged between 57% and 93% for different thrombus locations.

In renal artery thrombus, rtPA treatment resulted in CCR in one of two patients. rtPA was also recommended for bilateral RVT with evidence of renal impairment [19]. In a study of five newborns with unilateral RVT, clot resolution was not successful with rtPA plus heparin [20]. However, in another newborn, CCR was achieved when rtPA was used even 6 days after the onset of thrombus [21].

In three of four patients with stroke, PCR was achieved with thrombolysis. In the patient with left median cerebral artery thrombosis, PCR was achieved with rtPA administration even 12 h after diagnosis. Low rates of bleeding complications were reported with rtPA in cases of pediatric ischemic stroke [2,14], and reports about successful thrombolysis in pediatric stroke with systemic rtPA administered within the first 4 h are increasing [2,13,22,23].

For a 12-year-old patient with Behçet’s disease, rtPA was life-saving. Symptoms of thrombosis in multiple sites had developed within 15 days and systemic vasculitis was diagnosed. This patient was classified among the patients with pulmonary thromboembolism in this study, but he also had thrombi in the right atrium, vena cava inferior, bilateral iliac veins, vena porta, and vena hepatica. Administration of rtPA at 0.3 mg/kg/h for 46 h and concomitant UFH led to CCR. In this patient, there was major bleeding, which was controlled with temporary drug withdrawal and transfusion. There were four major non-fatal bleeding episodes (7.2%) in 55 systemic rtPA administrations. In addition to blood components, tranexamic acid may be used for bleeding complications. The use of antifibrinolytic agents for treating symptomatic intracranial hemorrhage that develops as a complication of thrombolytic therapy was also suggested [24]. Antifibrinolytics are available for urgent use compared to blood components.

In this series, CCR was achieved in only 13.3% of DVT episodes. In a pediatric study of 26 cases, there was no CCR in DVT, whereas CCR was achieved for 81% of arterial thrombi [10]. Successful thrombolysis in DVT with rtPA was reported in some pediatric studies [12,25]. In massive iliofemoral DVT with total occlusion of venous flow and/or compromise of arterial flow, there is a strong indication for systemic/catheter-directed (endovascular) thrombolysis or percutaneous mechanical thrombectomy to achieve venous patency [5]. Invasive interventions for thrombolysis of iliofemoral thrombosis are also technically possible in children aged 1-18 years and these approaches may reduce the risk or severity of PTS compared to standard anticoagulation alone [26]. Among the patients of the present study, endovascular thrombectomy was performed for one patient with iliofemoral thrombosis after unsuccessful systemic thrombolysis and extension of the thrombus to the contralateral extremity. However, thrombolysis with systemic rtPA was evaluated in this study.

In vitro studies show that rtPA has more rapid clot lysis activity and high affinity for fibrin, and it induces the binding of plasminogen to fibrin; due to these characteristics, it is recommended in newborns and children over other thrombolytics [19,27]. In the present study, alteplase was used with a half-life of 3-5 min; a short half-life provides bleeding control by transient drug withdrawal or decreasing the dose. UFH should be preferred for concomitant anticoagulation due to its short half-life. As a general practice, thrombolysis is attempted for vessel occlusions of up to 14 days due to the decreased response in cases of older thrombi [5]. Similar to our study, Ansah et al. [12] found no correlation between clot resolution and period from diagnosis to rtPA administration. In their study, the mean time from diagnosis to treatment was 36.0±16.8 h versus 18.1±5.3 h for CCR and PCR/NR, respectively.

### Study Limitations

There are limitations of this study due to its retrospective multicenter nature and the heterogeneity of the patient population. For some patients, thrombolysis was initiated 2 weeks after thrombus presentation. Participating centers generally had no standard protocols for thrombolysis. Only some centers used concomitant heparin, either in therapeutic or prophylactic doses. Imaging studies for diagnosis and monitoring were not standardized. Data on serum fibrinogen, fibrin degradation products, anti-factor Xa, prothrombin time, and activated partial thromboplastin time were not presented in this study. Thrombosis requiring thrombolysis is rare in children compared to adults and each center was already trying to develop its own experiences with thrombolysis during the 14-year study period. Prospective multicenter pediatric studies must be planned using standard protocols with the administration of continuous LD rtPA for 6-72 h and HD for not more than 6 h at a time.

## Conclusion

Systemic thrombolytic therapy with LD or HD rtPA may save lives and organs effectively if it is used for the right indications and at the right times in children with high-risk thrombosis. This therapy should be applied by experienced hematologists under close monitoring of recanalization and bleeding.

## Figures and Tables

**Table 1:**
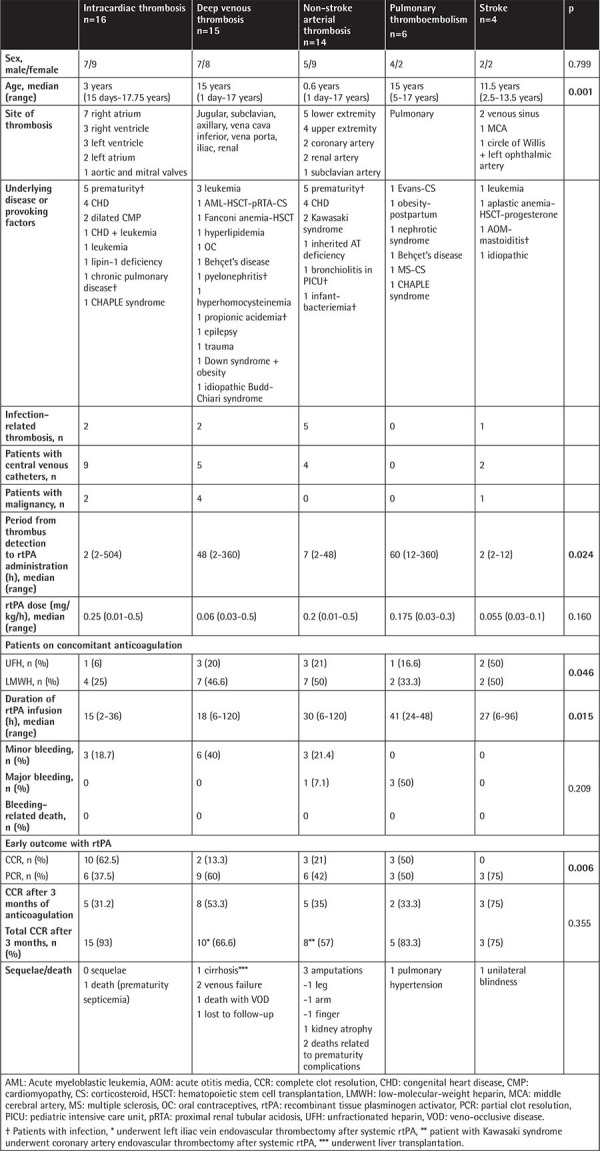
Characteristics of the patients and treatment outcomes with systemic recombinant tissue plasminogen activator.

**Table 2:**
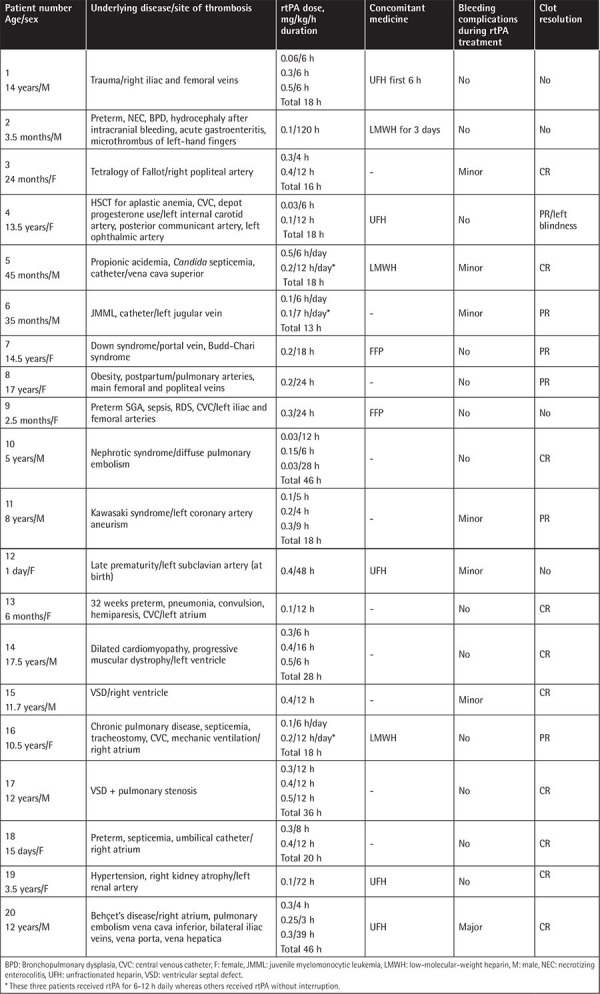
Outcomes and bleeding complications of patients using high-dose rtPA longer than 6 h/day.

**Table 3:**
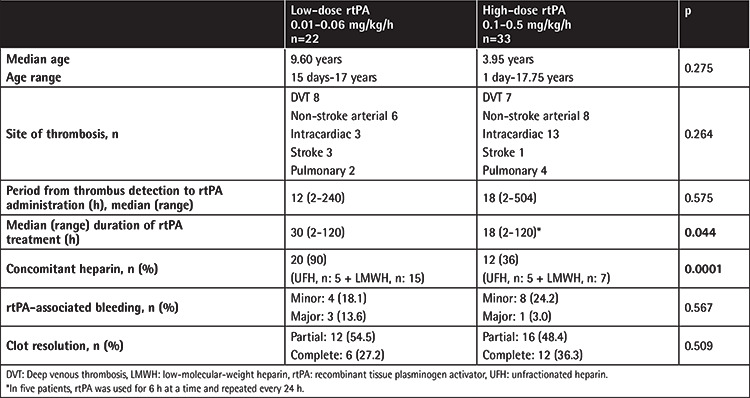
Comparison of systemic thrombolysis with low- and high-dose recombinant tissue plasminogen activator.
